# Lean body mass in living kidney donors impacts postoperative renal function

**DOI:** 10.1007/s00345-024-04908-3

**Published:** 2024-04-06

**Authors:** Robert A. Keenan, Aisling U. Nic an Riogh, David Brennan, Martina Morrin, Michael J. Lee, Niall F. Davis, Atakelet A. Ferede, Dilly M. Little

**Affiliations:** 1 Department of Surgical Affairs, Royal College of Surgeons, Dublin, Ireland; 2 National Kidney Transplant Service, Beaumont Hospital, Dublin, Ireland; 3 Department of Radiology, Beaumont Hospital, Dublin, Ireland

**Keywords:** Skeletal muscle mass, Sarcopenia, Kidney donation, Living kidney donation, Donor nephrectomy, Lean muscle mass, Kidney transplant

## Abstract

**Purpose:**

A living donor kidney transplant is the optimal treatment for chronic renal impairment. Our objective is to assess if lean skeletal muscle mass and donor factors such as body mass index, hypertension, and age impact on renal function following donor nephrectomy.

**Methods:**

Potential donors undergo CT angiography as part of their work-up in our institution. Using dedicated software (Horos®), standardized skeletal muscle area measured at the L3 vertebrae was calculated. When corrected for height, skeletal muscle index can be derived. Skeletal muscle mass index below predefined levels was classified as sarcopenic. The correlation of CT-derived skeletal muscle index and postoperative renal function at 12 months was assessed. Co-variables including donor gender, age, body mass index (BMI), and presence of pre-op hypertension were also assessed for their impact on postoperative renal function.

**Results:**

275 patients who underwent living donor nephrectomy over 10 years were included. Baseline pre-donation glomerular filtration rate (GFR) and renal function at one year post-op were similar between genders. 29% (*n* = 82) of patients met the criteria for CT-derived sarcopenia. Sarcopenic patients were more likely to have a higher GFR at one year post-op (69.3 vs 63.9 mL/min/1.73 m^2^, *p* < 0.001). The main factors impacting better renal function at one year were the presence of sarcopenia and younger age at donation.

**Conclusion:**

When selecting donors, this study highlights that patients with low skeletal mass are unlikely to underperform in terms of recovery of their renal function postoperatively at one year when compared to patients with normal muscle mass and should not be a barrier to kidney donation.

## Introduction

Kidney transplantation is the best treatment for chronic renal failure with living donation outperforming that of deceased donors [1]. Selection of patients for living kidney donation is crucial, however, for overall success and is not without its risks.

Serum creatinine, the most common universally measured indicator of renal function, is inherently and proportionally dependent on muscle metabolism and may be considered a surrogate for muscle mass [2, 3]. When adjusted for age and gender, creatinine can accurately estimate glomerular filtration rate (GFR) with the Chronic Kidney Disease Epidemiology Collaboration (CKD-EPI) being the most accurate formula for clinical use in well patients [2, 4–6].

Reduced skeletal muscle mass, a condition known as sarcopenia, is a normal part of aging, with an estimated 1% loss per year after the age of 30, and approximately 50% of people over the age of 80 years are expected to be deficient [7] This state may be seen in patients with malignancy, inflammatory diseases, and cardiac and renal disease, for example, and is independently associated with increased falls risk, increased morbidity in oncological surgeries, and higher all-cause mortality [8–14]. It is defined as calculated skeletal muscle mass more than two standard deviations below the expected muscle mass for normal healthy individuals [15]. Preexisting low skeletal muscle mass has been clearly linked to poorer outcomes in kidney transplant recipients in terms of increased hospital readmission posttransplant and mortality [16]. However, the effect of low muscle mass has not been assessed in the recovery of renal function in living kidney donors to date [3, 17].

Muscle mass has traditionally been measured with dual-energy X-ray absorptiometry (DEXA) scanning by measuring muscle bio-impedance; however, muscle mass measured at the L3 level on CT closely correlates with DEXA results, and CT has been shown to be a validated method of interrogating body composition which has been utilized in this study [18–20].

The aim of this study is to determine if pre-nephrectomy lean body mass index, body mass index, and other perioperative parameters impact on recovery of post-nephrectomy renal function in living kidney donors with the working hypothesis that sarcopenia (i.e., low lean muscle mass) negatively impacts on postoperative renal function recovery. This study differs from other studies assessing these factors as it assesses outcomes of healthy patients undergoing nephrectomy for an altruistic reason with no inherent underlying renal pathology.

## Methods

### Data collection

A retrospective review of a prospectively maintained database of all living kidney donor transplant pairs performed between January 1, 2010, and December 31, 2019, in the National Kidney Transplant Service, Beaumont Hospital, Dublin, Ireland, was undertaken. All donors were included in the initial data collection including open, laparoscopic, and hand-assisted laparoscopic donor nephrectomy approaches.

### Inclusion criteria

Anthropometric parameters including mass, height, body mass index, and patient sex were recorded in living kidney donors prior to donation and the presence of hypertension. Preoperative renal function, measured with serum creatinine concentration, was tested five to seven days prior to kidney donation. Postoperatively, serum creatinine at the first postoperative visit within six weeks and at routine follow-up at 1 year was recorded. For all creatinine measures, the corresponding estimated glomerular filtration rate (eGFR) was calculated according to the Chronic Kidney Disease Epidemiology Collaboration (CKD-EPI) equation which has been shown to be more accurate when compared to Modification of Diet in Renal Disease (MDRD) equations in healthy populations [4–6]. The equation is *A* × (serum creatinine/*B*)^*C*^ × 0.9938^age^ × (1.012 if female) × (1.159 if black) where *A* = 144 for females or 141 for males, *B* = 0.7 for females or 0.9 for males, and *C* = −0.339 for female with creatinine < /= 62 μmol/L and −1.209 if > 62 μmol/L or −0.411 if male with creatinine </= 80 μmol/L and −1.209 if creatinine > 80 μmol/L [21].

### Exclusion criteria

Patients were excluded if they did not have a complete data set required for analysis such as weight and height recorded prior to donation. CT angiograms that did not image the L3 plane were not included. Patients were excluded postoperatively if they did not have renal function measured within three and 12 months of donation or did not attend follow-up. Some patients may have met more than one exclusion criterion.

### Sarcopenia analysis

**Fig. 1:**
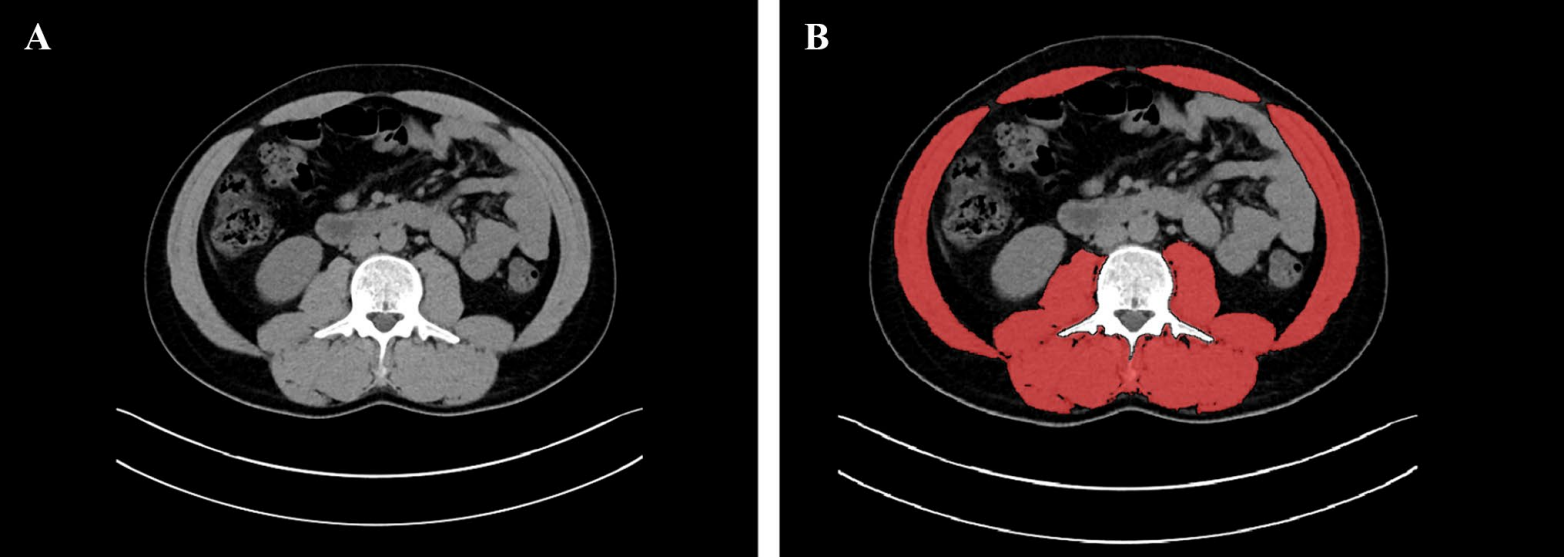
**A** Left—axial slice of non-contrast CT abdomen at the L3 vertebral level. **B** Right—all skeletal muscle at this level is highlighted in red

The skeletal muscle index for each patient was calculated as follows. All patients had preoperatively computed tomography (CT) abdominal angiography performed to assess renal vasculature. Attenuation levels of −29 to +150 were selected to calculate cross-sectional area of all visible skeletal muscle (including psoas, erector spinae, rectus abdominis, obliques, and quadratus lumborum) at the 3rd lumbar vertebrae (L3) on the patient’s CT using digital software, Horos (v3.3.6 Annapolis, MD USA) by 2 investigators (RK, AN) [22]. Figure 1 shows an example of skeletal muscle highlighted and calculated at the L3 vertebral level. The lean muscle mass area was corrected by height for calculation of the lean skeletal muscle mass index and expressed as a continuous variable. Values below 55.4cm^2^/m^2^ for males and 38.9cm^2^/m^2^ for females were selected to define low lean body mass index (i.e., sarcopenia), which equates to two standard deviations or less below the mean for normal healthy individuals [23]. Body mass index (BMI) was calculated and defined as overweight or not overweight according to the WHO criteria and expressed as a continuous variable [24]. Patient adverse outcomes of interest included GFR non-recovery, defined as GFR of < 60% of pre-donation GFR at 1 year.

### Statistical analysis

Student *t*-test, Pearson’s test, and Fisher’s exact tests were used to compare continuous variables and categorical variables as appropriate. Sarcopenia was assessed as a categorical value, while BMI was expressed as a continuous variable. The impact of the presence of sarcopenia and other variables and characteristics such as gender, age, hypertension, obesity, and pre-donation GFR on initial postoperative creatinine at six weeks and then at postoperative GFR at 1 year was evaluated using univariate and multivariate logistical regression analysis. A *p*-value of < 0.05 was considered statistically significant. Statistical analysis was performed using IBM SPSS for Statistics for Windows, version 25 (IBM Corp., Armonk, N.Y., USA).

## Results

### Baseline characteristics

Between January 1, 2010, and December 31, 2019, 358 living kidney donations were performed. 275 patients, 127 male and 148 female, who underwent living kidney donations were included in our final analysis following exclusions. 11 (4%) open nephrectomies and 264 (96%) laparoscopic/hand-assisted laparoscopic donor nephrectomies were performed although this difference had no statistical bearing on any outcome of interest.

The mean age at donation was 45.62 (range 23–72) years although female patients were statistically more likely to be older at 47.5 (± 10.1) years compared to male patients at 42.4 (± 10.5) years (*p* < 0.0001).

The mean length of stay was 4.8 days (± 1.2 days), 18.9% (*n* = 52) of patients had documented underlying hypertension, and the left kidney was used in 96.7% (*n* = 266) of cases without a statistically significant gender difference seen.

Male donors were more likely to have a higher BMI (26.6 kg/m^2^ ± 2.9 *vs* 24.9 kg/m^2^ ± 2.9, *p* < 0.0001) and more likely to be overweight (70.9%, *n* = 90 *vs* 46.6%, *n* = 69, *p* < 0.0001) than female donors. 33.9% (*n* = 43) and 26.4% (*n* = 39) of male and female patients, respectively, met the definition of sarcopenia based on their preoperative CT scans; however, this difference was not statistically significant.

Baseline renal function was similar between male (eGFR 99.2 mL/min/1.73 m^2^ ± 13.5) and female (eGFR 97.7 mL/min/1.73 m^2^ ± 13.9) patients prior to donation. No significant difference was seen in renal function at one year post-op between male patients (eGFR 66.1 mL/min/1.73 m^2^ ± 12.6) and female patients (eGFR 65.0 mL/min/1.73 m^2^ ± 13.4) nor in the proportion of patients displaying non-recovery of renal function (33.9%, *n* = 43 *vs* 26.4%, *n* = 39) (Table 1).

**Table 1:** Baseline characteristics of female and male patients. Female patients were more likely to be older and have a lower BMI

Variable	Female (*N* = 148)	Male (*N* = 127)	*p*-Value
Age, *m* (sd)	47.5 (10.1)	42.4 (10.5)	0.0001
Left kidney used, *n* (%)	144 (97.3)	122 (96.1)	0.737
Hypertension, *n* (%)	27 (18.2)	25 (19.7)	0.761
BMI, *m* (SD)	24.9 (2.9)	26.6 (2.9)	0.0001
Overweight, *n* (%)	69 (46.6)	90 (70.9)	0.0001
LOS, days, *m* (SD)	4.9 (1.3)	4.8 (1.3)	0.721
Baseline GFR, *m* (SD)	97.7 (13.9)	99.2 (13.5)	0.348
GFR at 12 months, *m* (SD)	65.0 (13.4)	66.1 (12.6)	0.484
Sarcopenia, *n* (%)	39 (26.4)	43 (33.9)	0.175

### Impact of sarcopenia

By our criteria, *n* = 82 (29.8%) of all donors met the criteria for sarcopenia. Gender, age, and underlying presence of hypertension were not statistically significantly different between the sarcopenic vs non-sarcopenic groups. The mean BMI for sarcopenic patients (24.5 kg/m^2^ ± 2.9) was statistically lower than their non-sarcopenic counterparts (26.2 kg/m^2^ ± 3.0, *p* < 0.0001). 43.9% (*n* = 36) *vs* 63.8% (*n* = 123) of sarcopenic and non-sarcopenic patients, respectively, met the WHO criteria for being overweight based on BMI.

Baseline pre-donation renal function was similar between both groups (100.61 mL/min/1.73 m^2^ ± 12.8 *vs* 97.31 mL/min/1.73 m^2^ ± 14.0) although sarcopenic patients did have a statistically significantly better GFR at 12 months (69.3 mL/min/1.73 m^2^ ± 12.1) post-donation compared to non-sarcopenic donors (63.9 mL/min/1.73 m^2^ ± 13.1). A higher proportion, 25.4% (*n* = 49), of non-sarcopenic patients did not recover their renal function compared to only 13.4% (*n* = 11) which was significant (*p* < 0.037) (Table 2).

**Table 2:** Anthropometric and clinical baseline characteristics of sarcopenic vs non-sarcopenic kidney donors. BMI was statistically higher in the non-sarcopenic group, and they had a lower GFR at 12 months

Variable	Sarcopenia (*N* = 82)	Non-sarcopenia (*N* = 193)	*p*-value
Female sex, *N* (%)	39 (47.6)	110 (56.9)	0.175
Age, *m* (sd)	46.0 (11.5)	44.8 (10.2)	0.371
Left kidney used, *n* (%)	80 (97.6)	186 (96.4)	0.999
Hypertension, *n* (%)	18 (22.0)	34 (17.6)	0.401
BMI, *m* (SD)	24.5 (2.9)	26.2 (3.0)	0.0001
Overweight, *n* (%)	36 (43.9)	123 (63.8)	0.002
Baseline GFR, *m* (SD)	100.6 (12.8)	97.3 (14.0)	0.079
GFR at 12 months, *m* (SD)	69.3 (12.1)	63.9 (13.1)	0.001
GFR recovery < 60% at 12 months, *N* (%)	11 (13.4)	49 (25.4)	0.037

### Renal function at 1 year

The largest change in GFR at one year post-donation in our study group was a reduction of 58%. The mean GFR at one year post-donation was 64.9% ± 9.15% when expressed as a percentage of the pre-donation GFR. Following multivariable linear regression analysis, patients who recovered their renal function at 12 months following their donation were more likely to be younger (*p* < 0.0001), have higher pre-donation GFR (*p* < 0.0001), and more likely to have sarcopenia (*p* < 0.001). Gender, kidney laterality, presence of underlying hypertension, and BMI had no statistical bearing on recovery of renal function (Table 3).

**Table 3:** Multivariable associations with the recovery of GFR at 1 year post-donation. Younger patients, those with sarcopenia and those with a higher starting GFR, were more likely to have a better GFR at 12 months post-op

Variable	Hazard ratio	95% CI	*p*-Value
Gender (female)	1.753	−3.909 to 0.403	0.111
Age	−0.412	−0.529 to −0.296	0.0001
Hypertension	−1.81	−0.433 to 0.719	0.159
BMI	−0.256	−0.606 to 0.094	0.151
Sarcopenia	4.076	1.794 to 6.359	0.001
Pre-donation GFR	0.496	0.410 to 0.583	0.0001

## Discussion

The performance of living kidney donation exceeds that of deceased donors in terms of graft and patient survival [1]. Therefore, elective living donor nephrectomy is the preferred method of transplantation where possible [1]. 214 living donor kidney transplants were performed in Ireland between 2014 and 2019, averaging 43 per year in that time [1]. Graft and recipient patient survival remains excellent at 95% and 100%, respectively, at five years. No fatalities or chronic renal failure requiring dialysis has occurred in any donor related to their kidney donation since the commencement of the program in 2009 [1]. In keeping with international standards, mortality within the donor group is lower than general population which is reassuring for clinicians when consenting to any potential donors [25].

The effect of radiologically calculated skeletal muscle index and sarcopenia on postoperative outcomes has been examined in a number of large studies including urological and gastrointestinal cancer operations; however, this appears to be the first study assessing the impact in otherwise healthy patients. In major oncological operations, sarcopenia has been implicated in increased postoperative complications and reduced overall and cancer-specific survival following pancreatic resection and hepatectomy for hepatocellular carcinoma (HCC) and radical cystectomy for urothelial malignancy [8, 12, 14]. A systematic review and meta-analysis looking at skeletal muscle index prior to treatment for a range of solid organ malignancies showed significance in overall survival (hazard ratio 1.44), cancer-specific survival (HR 1.93), and disease-specific survival (HR 1.16) although not progression-free survival associated with the presence of sarcopenia [13]. This paper, however, included a large number of studies of patients with advanced or metastatic cancers including pancreatic, HCC, and colorectal tumors where muscle wastage can occur due to the disease itself and not solely due to treatment. Conversely, sarcopenia appears to have no impact on the oncological or functional outcome of men following radical prostatectomy [26].

When looking specifically at surgery for renal tumors, reduced skeletal muscle index measured within 30 days prior to nephrectomy for localized non-metastatic renal cell carcinoma (RCC) is independently linearly associated with increased 5-year cancer-specific mortality (HR 1.7) and all-cause mortality [11]. A separate study assessing sarcopenic vs non-sarcopenic patients undergoing nephrectomy for stage III/IV RCC did not find any statistically significant difference in overall specific survival between the groups although authors did postulate that the survival may be more influenced by the natural history of advanced RCC rather than the degree of muscle wastage [10]. The major confounder between these studies and our study here is of course the presence of malignancy. When assessing the value of skeletal muscle index in noncancer surgery, the literature is much less populated. Researchers in Japan have shown that renal donors with elevated CT-measured abdominal visceral fat are more likely to develop chronic renal impairment at 12 months postoperatively, while a separate group has demonstrated predictability of renal function following donation based on a ratio of visceral-to-subcutaneous fat measured on CT [27]. The data presented here within counterintuitively shows that patients with sarcopenia were in fact more likely to recover renal function and have a higher GFR one year following kidney donation. Reasons for this are unclear; however, the pre-nephrectomy GFR was higher in the sarcopenic population although this was nonstatistically significant.

The development of ESKD in donors is rare. In our study, no patient developed renal impairment worse than stage 3b (GFR < 30 mL/min/1.72 m^2^) at one year although long-term data have not been assessed. In a study assessing change in eGFR in the donor population compared to a matched non-donor population, the eGFR in the donor pool will increase (likely due to compensatory hypertrophy); however, this will plateau by five years post-op. Reassuringly, one study from Nam et al. looking at Canadian donors showed that mean eGFR ten years following kidney donation remains > 60 mL/min/1.72 m^2^ in almost all donors [28]. Overall absolute risk of developing ESKD has been shown in several studies, however, to be marginally increased compared to matched non-donor population [29, 30]. Studies looking at the long-term outcomes following living kidney donation are rare although one from Matas et al. looking at over 4000 donors dating from 1963 to 2015 identified only 39 (0.01%) cases of ESRD, diagnosed 27 years following donation with male sex, younger age at donation, and smoking implicated as risk factors for developing ESRD [31]. It can therefore be postulated that developing CKD purely based on kidney donation is particularly rare.

In our study, increasing age was also associated with a decreased likelihood of optimal recovery of renal function. This is validated by a large meta-analysis of living kidney donors which identified a larger GFR decline with age > 60 years and inferior outcomes in obese and male patients [32]. Although BMI and obesity were not statistically associated with worse renal outcome in our patients on multivariate analysis, these factors were implicated in Bellini et al.’s meta-analysis [32]. Okumura et al. have developed a prediction tool for eGFR and compensatory renal hypertrophy following donation based on gender, age, history of hypertension, pre-donation eGFR, and remaining kidney volume relative to body weight which, when modeled, correlates strongly with eGFR following donation [33]. Further studies based on Japanese donors looking at donor body mass index (BMI) and preserved kidney volume adjusted for body surface area found these variables to be predictive of unfavorable renal function compensation (defined as a decline in eGFR of > 30% at one year post-op) following donation.

From our results, we can conclude that sarcopenia, a condition of reduced skeletal muscle mass, and younger age are statistically associated with an increased likelihood of optimal recovery of renal function following a living donor nephrectomy at one year. This is relevant when assessing patients who come forward as potential kidney donors. As part of the consent process, the presence of low skeletal muscle mass should not be considered a barrier to proceeding with donor nephrectomy based on our results. Furthermore, younger patients may be preferred; however, the overall statistical impact of increasing age (OR 1.067) may in fact be negligible clinically, thus reassuring that older donors may also be considered in the knowledge that age plays a minor role in determining subsequent renal function.

### Limitations

All donors in our institution undergo a nuclear medicine scan to assess renal function prior to donation; however, this is not performed postoperatively; therefore, measured serum creatinine and calculated GFR must be used for comparison before and after nephrectomy. Many methods exist for calculating GFR including the Cockroft–Gault equation which incorporates weight in the calculation. As weight can fluctuate, we decided to omit the weight-based GFR calculation, so it is only accounted for in the skeletal muscle index calculation although this may not be generalizable across a heterogeneous population. We have not statistically assessed other surgical and perioperative factors that may affect renal function such as vascular complexity or operative duration. This study assumes no considerable medical insults that may otherwise impact renal function have affected the patient in the first postoperative year. Furthermore, this study does not assess the long-term impact of sarcopenia on renal function beyond one year.

## Conclusion

In this study, we have assessed baseline characteristics of patients undergoing living donor nephrectomy in a national transplant center in particular focusing on anthropometric factors such as weight, body mass index, and skeletal muscle index calculated by CT and the impact of these factors on the recovery of renal function at one year postoperatively. Based on our findings, younger patients and those with lower skeletal muscle mass are statistically more likely to have a better renal function at one year post-kidney donation. This information may be beneficial when counseling potential donors, in particular borderline patients or those with some underlying renal impairment to begin with. Overall, however, donors can be reassured that the overall impact on their renal function at one year post-op is an expected reduction of 35%. Donor nephrectomy in our institution has been demonstrated to be safe with excellent and safe outcomes for the donor.

## Data Availability

The data that support the findings of this study are available from the corresponding author, RAK, upon reasonable request.

## Abbreviations

BMI: Body mass index
CKD-EPI: Chronic Kidney Disease Epidemiology Collaboration
CT: Computed tomography
DEXA: Dual-energy X-ray absorptiometry
ESKD: End-stage kidney disease
GFR/eGFR: Estimated glomerular filtration rate
HCC: Hepatocellular carcinoma
MDRD: Modification of diet in renal disease
RCC: Renal cell carcinoma
WHO: World Health Organization
